# Binocular rivalry produced by temporal frequency differences

**DOI:** 10.3389/fnhum.2012.00227

**Published:** 2012-07-31

**Authors:** David Alais, Amanda Parker

**Affiliations:** School of Psychology, The University of SydneySydney, NSW, Australia

**Keywords:** binocular rivalry, temporal channels, temporal frequency, form, motion

## Abstract

When the eyes view images that are sufficiently different to prevent binocular fusion, binocular rivalry occurs and the images are seen sequentially in a stochastic alternation. Here we examine whether temporal frequency differences will trigger binocular rivalry by presenting two dynamic random-pixel arrays that are spatially matched but which modulate temporally at two different rates. We found that binocular rivalry between the two temporal frequencies did indeed occur, provided the frequencies were sufficiently different. Differences greater than two octaves (i.e., a factor of four) produced robust rivalry with clear-cut alternations similar to those experienced with spatial rivalry and with similar alternation rates. This finding indicates that temporal information can produce binocular rivalry in the absence of spatial conflict and is discussed in terms of rivalry requiring conflict between temporal channels.

## Introduction

Binocular rivalry occurs when two sufficiently different images are presented to each eye. This prevents binocular fusion of the two images and triggers a stochastic alternation between the monocular images (Blake and Logothetis, 2002; Alais and Blake, 2005; Alais, 2011). This perceptual alternation is of considerable interest to visual neuroscientists because despite two distinct images entering the visual system, only one of them reaches conscious perception. Generally, binocular rivalry is induced by presenting pairs of images that differ in terms of a spatial property, typically orientation, but rivalry can also be triggered by interocular differences in spatial frequency, form and color (Yang et al., 1992; Kovacs et al., 1996; Tong et al., 1998; Alais and Melcher, 2007). In this paper, we will focus on the temporal dimension and examine whether binocular rivalry can be elicited by interocular differences temporal frequency differences.

The easiest way to introduce temporal modulations is to use motion and it has long been known that motion can greatly influence rivalry. For example, if one stimulus is set in motion, it will strongly predominate over a static pattern (Breese, 1899; Walker and Powell, 1979; Blake et al., 1985). Flickering a rival target too will enhance its predominance over the other target (Blake and Fox, 1974). Rivalry will also occur when both rival targets are motion stimuli, provided they drift in different directions or at different speeds (Fox et al., 1975; Wade et al., 1984; Blake et al., 1985; Wiesenfelder and Blake, 1990; Alais and Blake, 1998; Blake et al., 1998; Nguyen et al., 2003; Alais and Parker, 2006). A general limitation of this literature is that form and motion are often confounded because the motion stimuli also differ in spatial form (e.g., drifting orthogonal gratings). A further problem is that motion is a step removed from the basic mechanisms of temporal processing, since visual temporal filters logically precede the computation of speed and direction (Reisbeck and Gegenfurtner, 1999; Priebe et al., 2006).

Orthogonally oriented drifting gratings are commonly used to elicit motion rivalry (Alais and Blake, 1998; Andrews and Blakemore, 2002). With such stimuli, it could well be the orientation conflict that is responsible for initiating rivalry, rather than the motion. Similarly, form differences between opposite-throw spirals (Nguyen et al., 2003) and radial versus concentric patterns (Wade et al., 1984) could provide the image conflict that provokes rivalry rather motion conflict. The same can be said of orthogonally drifting random-dot patterns (Blake et al., 1998; van de Grind et al., 2001) because translating random-dot patterns create motion streaks (Geisler, 1999) when drifting fast, effectively transforming them into a type of grating. Recent studies have confirmed that “motion streaks” created by translating random-dot patterns do activate orientation-selective mechanisms (Apthorp et al., 2010, 2011) and do produce an orientation-specific suppression in binocular rivalry (Apthorp et al., 2009).

The seeming inevitability of the form/motion confound has led some researchers to conclude that it is form conflict that triggers rivalry and that rivalry between motion signals does not occur at all (Ramachandran, 1991; He et al., 2005). It is worthwhile resolving this question because if rivalry can occur between temporal modulations, then the temporal dimension must have an input into the binocular matching process. To verify this would require rival stimuli that differ only in the temporal dimension and which still elicit rivalry alternations. One attempt to do this tested whether rivalry would occur between motion aftereffects produced by adaptation to orthogonal translating gratings (Blake et al., 1998). Testing the aftereffects with a binocularly-viewed dynamic test pattern did elicit rivalry alternations. Against this, however, another study using full-field flicker found that different temporal modulation rates in each eye failed to elicit any rivalry at all (O'shea and Blake, 1986).

Complicating the debate further are findings showing that motion and form can rival independently (Andrews and Blakemore, 1999; Alais and Parker, 2006). In Andrews and Blakemore's study, orthogonally drifting gratings were presented dichoptically and it was found that the two orientations rivaled reliably but the motions did not. On about 50% of trials, the single grating that happened to predominate at a given moment did not drift orthogonally to its orientation but obliquely—in the direction expected if both motions were integrated (inconsistent with one motion being suppressed). Similar results have been reported by another group (Cobo-Lewis et al., 2000). It has also been found that overlaying two orthogonally drifting gratings of low spatial frequency and viewing them through a binocular grid (allowing a fine-scale binocular match) will completely prevent rivalry from occurring (Carlson and He, 2004). In such a case, a dichoptic plaid is perceived through the apertures of the grid which moves in the global motion direction defined by the “intersection of constraints” rule (Adelson and Movshon, 1982; Alais et al., 1994).

Overall, it is not clear from the literature whether interocular temporal frequency differences elicit rivalry. The presence of form conflict clearly represents a confound in many rivalry studies using motion stimuli, and using motion to assess the role of temporal frequency is not the most direct approach. An ideal stimulus would contain temporal modulations and no form conflict. The full-field flicker stimulus of O'shea and Blake (1986) comes close to this, but it contains no contrast—the primary attribute driving the response level of early visual neurons. In the present study, we will examine whether interocular temporal differences elicit rivalry using a novel stimulus: a random dynamic-noise sequence that is temporally filtered into narrow temporal pass-bands. Being spatially random, the stimulus contains no coherent form to confound the results and it modulates temporally without translating in any direction, removing the motion direction confound. It also contains visual contrast to effectively drive visual neurons and allows precise control over temporal frequency, with the advantage that spatial frequency can be filtered independently.

To preview the results, we find that interocular temporal frequency differences do elicit rivalry alternations—very reliably for differences greater than two octaves (Experiment 1)—and rivalry alternations experienced for large temporal frequency differences have a similar character to those elicited in spatial rivalry (e.g., orthogonal gratings), with perceptual alternations occurring crisply every two seconds or so. When the modulation rates are too close to engage in rivalry, observers perceive the average temporal frequency and do not perceive temporal beating at the difference frequency (Experiment 2). When the modulation rates do differ enough to produce robust rivalry, observers can accurately select the perceptually alternating frequencies from a range of non-rivaling comparison frequencies (Experiment 3). Finally, we show that measures of alternation dynamics for robust temporal frequency rivalry are comparable to those of spatial rivalry (Experiment 4).

## General methods

### Subjects

The first two authors served as subjects in all experiments, together with two or three naïve observers. All had normal stereo acuity and normal or corrected visual acuity.

### Stimuli

**Figure 1:**
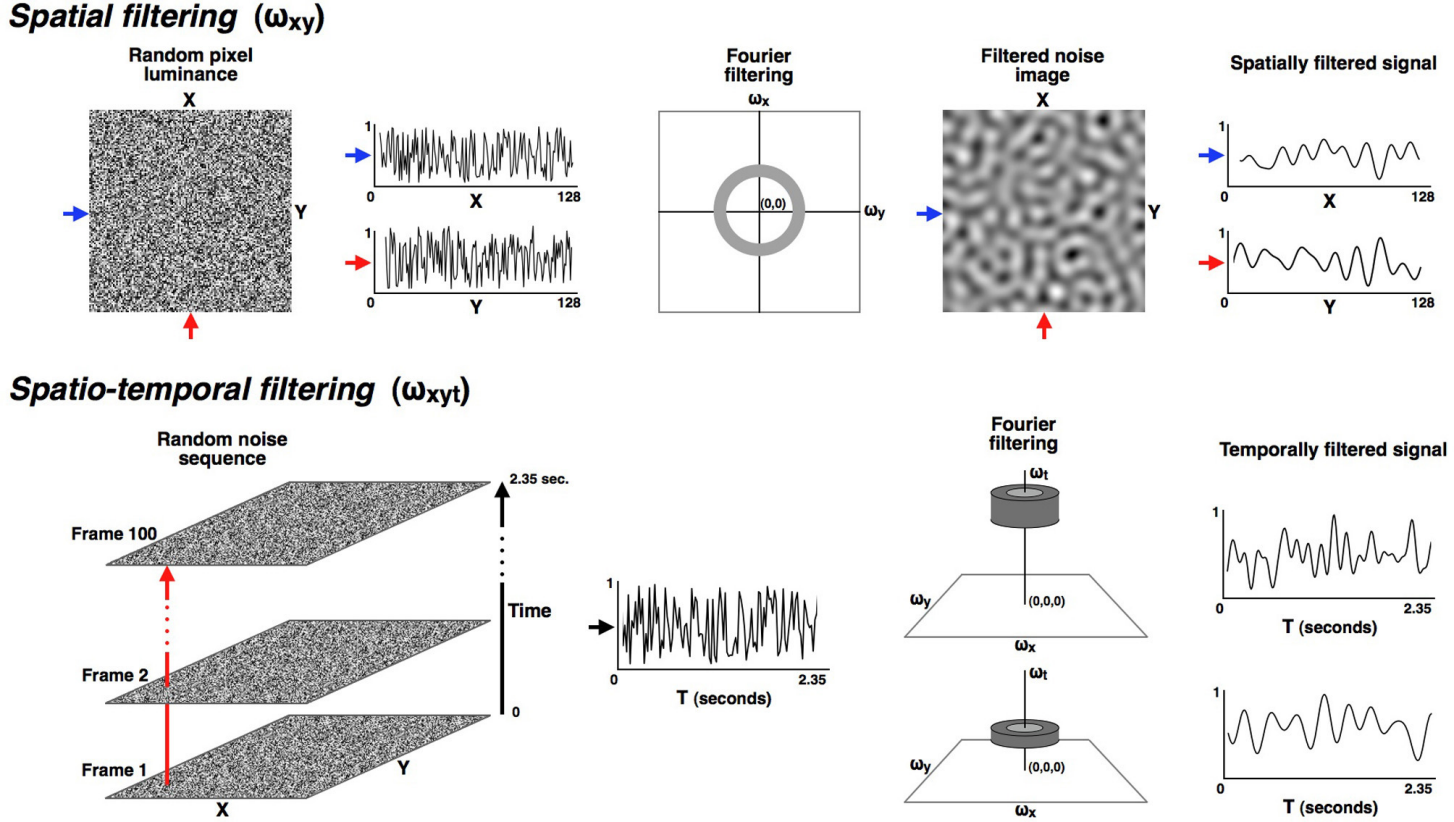
**All stimuli were made from a random dynamic noise sequence that was filtered in both the spatial and temporal dimensions into narrowly defined pass-bands.** The upper part of the figure illustrates the spatial filtering. All stimuli in these experiments were isotropically filtered into a fixed spatial pass-band ranging from 0.8 to 1.6 cyc/deg. The lower row illustrates how the noise sequences were filtered in the temporal dimension so that the modulation rate over time could be carefully controlled. Six narrow temporal pass-bands were used in these experiments, with each band having a full-width of 0.33 octaves (where octaves are calculated as the base-two logarithm of the ratio of the upper and lower cut-off frequencies). The pass-bands were all separated by one octave and had center frequencies of: 0.59, 1.18, 2.37, 4.73, 9.47, and 18.93 Hz. Since the time dimension is orthogonal to the (x, y) plane it could be manipulated without altering the spatial frequency range, which remained constant at 0.8 to 1.6 cyc/deg for all conditions.

To make the temporally filtered random dynamic dot sequences (see Figure 1), 100 random-dot noise patterns were generated. Each noise pattern was 128 by 128 pixels with a 2-pixel check size and subtended 2.5° of visual angle at the viewing distance of 57 cm. Playing these images as an animation would produce standard dynamic random noise with a very broad (white) frequency spectrum. Our approach in this paper was to filter these image sequences in the temporal frequency domain to produce narrow bands of temporal frequencies. Before temporal filtering, the stack of 100 noise images was duplicated so that the left and right eyes received spatially identical noise sequences. The image stack was then Fourier transformed and filtered in frequency space using a three-dimensional mask (x, y, t) in which the height of the image stack (100 images, in this case) represents the time dimension. The video monitor had a vertical scan rate of 85 Hz and noise images were updated every second refresh to produce an image update rate of 42.5 Hz and therefore a maximum achievable temporal frequency of 21.25 Hz.

The available temporal frequency range of 0 to 21.25 Hz was filtered into six narrow temporal pass-bands, each with a full-width of 0.33 octaves. The pass-bands were octave multiples of each other and had center frequencies of: 0.59, 1.18, 2.37, 4.73, 9.47 and 18.93 Hz. Since the time dimension is orthogonal to the (x, y) plane, spatial, and temporal dimensions could be filtered independently. The spatial filtering for all conditions in these experiments was band-pass with a full-width of 1 octave and a center frequency of 1.13 cyc/deg so that the only difference between left- and right-eye stimuli was temporal frequency. After spatiotemporal filtering the images were back transformed from the frequency domain and normalized to the full luminance range of the monitor to maximize stimulus contrast.

All stimuli were generated using the psychophysics toolbox (Brainard, 1997; Pelli, 1997) for Matlab on a G4 Macintosh computer and were presented on a 22″ Phillips CRT monitor (1024 × 768 resolution) with a refresh rate of 85 Hz. Stimuli were viewed through a mirror stereoscope, with a black square frame surrounding the circular stimulus apertures to aid binocular fusion. A small fixation cross was positioned in the center of the stimuli to help minimize eye movements. The average luminance of the stimulus arrays was 34.7 cd/m2 and the background region of the monitor was set to this value.

## Experiment 1

The aim of the first experiment was twofold: to determine whether dichoptic, spatially matched stimuli modulating at different temporal rates elicit rivalry alternations, and to find which frequency pairs rival most vigorously. Subjects therefore observed all pairings of the six temporal frequencies for 15 s and indicated whether at least one rivalry alternation was perceived.

Before the experiment we ran a pilot to determine whether the various temporal frequency pairings needed to be equated for stimulus strength (Levelt, 1965). An earlier rivalry study using uniform fields of flicker showed that high temporal frequencies tend to predominate over lower ones (O'shea and Blake, 1986). We therefore measured predominance ratios of various temporal frequency combinations at four different contrast levels. If a strong tendency for high-frequency stimuli to predominate is observed, reducing its contrast will be an effective means to weaken it and equate the two stimuli.

### Methods

**Figure 2:**
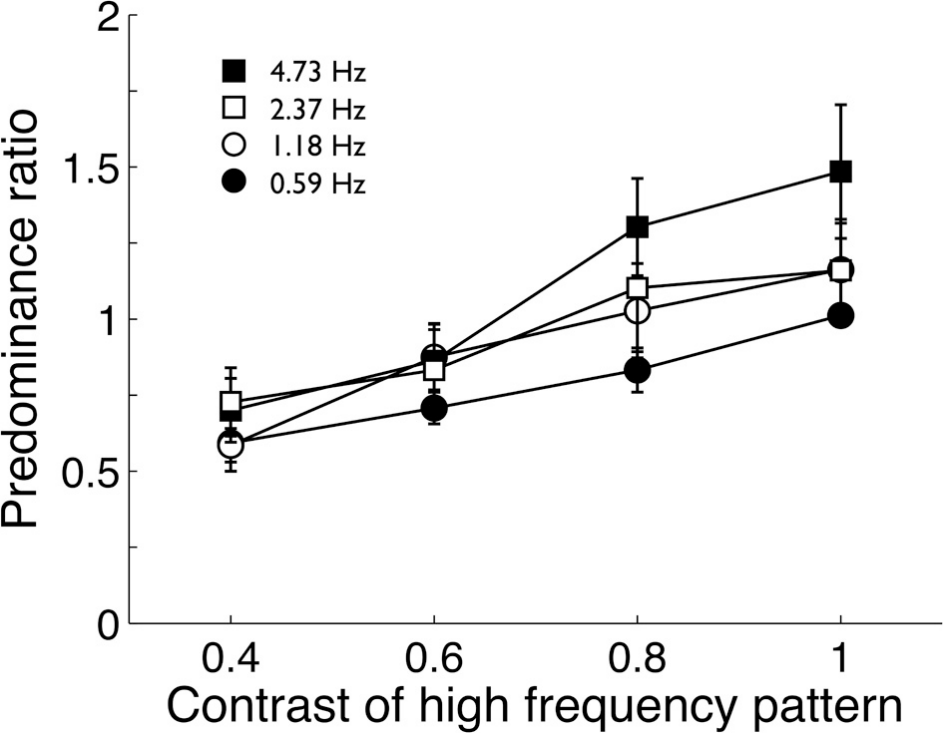
**Pilot data (group mean; *n* = 4) from Experiment 1 showing the predominance ratio of a high temporal frequency pattern (18.93 Hz) to various lower temporal frequency patterns.** The tendency of the high frequency pattern to predominate over lower frequencies declined as its contrast was reduced. Error bars show ±1 standard error of the mean.

The pilot experiment used the fastest modulation (18.93 Hz) paired with four slower modulations (4.73, 2.37, 1.18, and 0.59 Hz). Four observers viewed the four stimulus pairs for 2 min while tracking perceptual alternations between fast and slow modulations. Observers did three repetitions of each pair and predominance ratios (total time the fast image was visible divided by the total time the slow image was visible) were averaged. Group means are shown in Figure 2. Observers repeated this procedure four times with the high temporal frequency stimulus (18.93 Hz) taking one of 4 contrast levels (0.4, 0.6, 0.8, and 1.0) in a randomized order. The lower frequency stimuli were fixed at maximum contrast.

The stimuli in Experiment 1 are described in General Methods and Figure 1. Participants did four sessions of 105 trials each. In each session, the full set of 15 frequency combinations was presented, plus six kinds of catch trial (one for each of the 6 temporal frequencies) in which identical temporal frequencies were paired to verify that participants were responding correctly to perceived alternations. These 21 stimulus combinations were repeated five times in a session, with each session repeated four times. Trials were self-paced and the order within a session was completely randomized. Participants were instructed to press a key if they saw a perceptual alternation in modulation rate (from fast to slow, or vice versa). If a key was pressed to indicate rivalry, participants were taken straight to the next trial, otherwise the trial continued for the full 15 s.

We also ran a control condition to see whether the static frames would elicit rivalry. The reason is that although the left- and right-eye patterns are made from matched noise patterns, once temporally filtered they modulate at different rates and the relative phase between them varies periodically. Using the same method just described, five subjects made 16 judgments of rivalry incidence for static images selected to have a phase difference of either 90°, where the modulations are orthogonal (i.e., independent), or 180°, where the patterns are maximally different (i.e., anti-phase). On each trial, one eye viewed a frame selected at random from the modulation sequence and the other viewed a subsequent frame corresponding to either 90° or 180° phase offset.

### Results: pilot data

Pilot data showed the high-frequency stimulus did tend to predominate over the lower frequency patterns when both had maximum contrast (Figure 2). As expected, reducing the contrast of the high-frequency stimulus reduced it predominance, confirming O'Shea and Blake's (1986) finding. At maximum contrast, none of the stimulus pairs produced extremely biased predominance ratios and none were greater than 2:1 and it was decided to maintain all stimuli at maximum contrast.

### Results: Experiment 1

The data from Experiment 1 are shown in Table 1 which shows the incidence of rivalry for each temporal frequency pair. The dark oblique shows cells with a temporal frequency difference of one octave, and the light oblique shows a three-octave difference. Temporal frequency pairs on or above the light shaded oblique (i.e., three-, four-, and five-octave differences) all produced reliable rivalry alternations. The average rivalry incidence for a three-octave difference was 0.87, and ~1.0 for four- and five-octave differences. Overall, rivalry incidence increased strongly with temporal frequency difference *F*_(4, 12)_ = 89.325, *p* < 0.001. As the data on the major oblique show, subjects never falsely reported rivalry alternations on the catch trials.

**Table 1:** **Proportion of trials yielding a perception of binocular rivalry for various temporal frequency combinations**.

		**Temporal frequency 1 (Hz)**					
		**0.59**	**1.18**	**2.37**	**4.73**	**9.47**	**18.93**
**Temporal**	**0.59**	0	0.01	0.13	0.69	0.98	1.00
**frequency 2**	**1.18**		0	0.05	0.43	0.95	1.00
**(Hz)**	**2.37**			0	0.15	0.89	0.98
	**4.73**				0	0.23	0.87
	**9.47**					0	0.11
	**18.93**						0

Data are group means averaged across five observers. Along each diagonal, the cells are equally separated in temporal frequency when expressed in terms of octave differences (i.e., the ratio of the two frequencies expressed as a base-two logarithm). The dark gray shading shows frequency combinations differing by one octave, and the light gray shading represents frequency combinations differing by three octaves.

**Figure 3:**
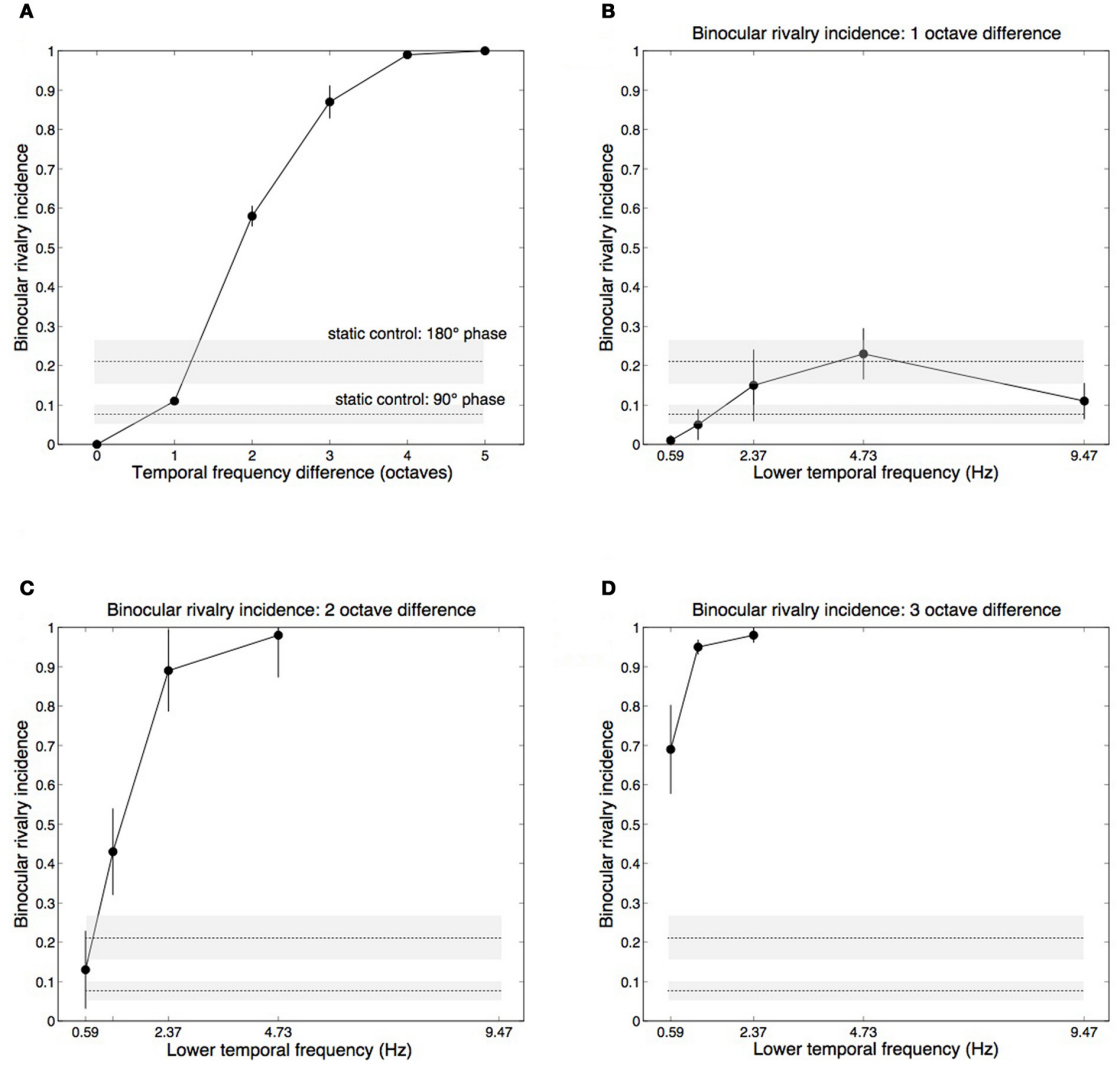
**Group mean data with ±1 standard errors from Experiment 1 showing rivalry incidence for various temporal frequency combinations.** Horizontal dashed lines show results from a static control condition (see Methods), with ±1 standard error shading. **(A)** Rivalry incidence increases with temporal frequency difference. The data here are means of the diagonals in Table 1. **(B)** Rivalry incidence for one-octave temporal frequency differences. The x-axis shows the lower frequency of the two rivaling frequencies. One-octave differences are generally not sufficient to trigger rivalry. **(C)** Two-octave frequency differences produce robust rivalry except at the low end of the temporal dimension. **(D)** Three-octave frequency differences produce robust rivalry at any point of the temporal dimension.

The main effect of temporal frequency difference on rivalry incidence (average of each oblique in Table 1) is shown in Figure 3A. Contrasts testing for trends revealed a significant linear trend, [*F*_(1, 3)_ = 209.69, *p* = 0.001], and quadratic trend, [*F*_(1, 3)_ = 62.108, *p* = 0.004]. Figures 3B,C,D plot the one-, two-, and three-octave obliques from Table 1, showing that the effectiveness of a given temporal frequency difference in eliciting rivalry varies along the temporal frequency dimension. The effect of temporal frequency difference was significant for two octaves, [*F*_(3, 12)_ = 33.732, *p* < 0.001] (Figure 3C) and three octaves, [*F*_(2, 8)_ = 5.580, *p* < 0.05] (Figure 3D). Significance in this case indicates rivalry incidence for a given difference depends on the frequencies making up the pair. Figures 3C,D show that a given frequency difference is more effective when located at the higher end of the frequency spectrum. There were no significant effects for the one-octave difference, [*F*_(4, 16)_ = 2.530, *p* = 0.081], or four-octave difference, [*F*_(1, 4)_ = 2.667, *p* = 0.178].

Although each eye's temporal modulation were made from identical noise images, they modulate at different rates and so their relative phases vary over time. The horizontal lines in Figure 3 plot group mean results from the control conditions in which we measured rivalry incidence for two static images taken from different points of the modulation sequence to determine if phase differences contribute to temporal frequency rivalry. We chose two phase offsets, 90° (where the motion sequences are independent, in cosine and sine phase) and 180° (where the sequences are in anti-phase and are maximally different). Sustained viewing for 15 s of the static phase differences did produce alternations, although much less than the two- and three-octave conditions that rivaled vigorously (Figures 3C,D).

### Discussion

These data establish that differences in temporal modulation rates between spatially matched patterns do indeed engage in binocular rivalry. Overall, the most straightforward summary of the data is that a temporal frequency difference of at least two octaves is needed to generate temporal frequency rivalry (Figure 3A). This is surprisingly large, especially when compared to rivalry between spatial frequency or orientation differences. This probably reflects the fact that there are only two (or three) temporal frequency channels in the visual system (Mandler and Makous, 1984; Anderson and Burr, 1985; Hammett and Smith, 1992; Hess and Snowden, 1992; Cass and Alais, 2006), whereas spatial frequency and orientation channels are more numerous, perhaps numbering six to eight (Graham, 1972; Stromeyer and Julesz, 1972; Braddick et al., 1978; Graham, 1989), and are therefore more tightly tuned than temporal frequency channels. This is taken up in the “General Discussion,” but the clear implication is that rivalry occurs when the stimuli are sufficiently different to activate separate channels. In the temporal domain, this requires a rather large difference of about two octaves, whereas the narrower orientation and spatial frequency channels require only a one-octave difference to produce rivalry (Blakemore, 1970; Braddick et al., 1978; Yang et al., 1992).

Although a two-octave temporal frequency difference will generally elicit binocular rivalry, sheer frequency difference does not entirely explain the data in Table 1. There is a dependence on where a given frequency difference is located along the temporal frequency dimension. Looking at Figure 3C, the higher frequency pairings were more likely to produce rivalry alternations. This tendency is also present in the three-octave data (Figure 3D) where rivalry was less likely for 4.73 vs. 0.59 Hz than for the same frequency difference located higher on the temporal frequency dimension. This interaction most likely arises from the location and intersection point of the underlying temporal channels.

Most investigations of temporal channels have revealed a broad, low-pass channel at the low end of the frequency spectrum with a band-pass channel at the high end (Anderson and Burr, 1985; Hess and Snowden, 1992; Snowden et al., 1995; Cass et al., 2009b). There is also some evidence for a second, higher band-pass filter in a three-channel model (Mandler and Makous, 1984; Hess and Snowden, 1992; Johnston and Clifford, 1995). In either case, the low-pass channel crosses over the high bandpass channel at about 6–8 Hz. Table 1 shows that rivalry incidence is highest when the two frequencies span this crossover point. This is true for the four- and five-octave differences (which average 0.99 incidence), and for the two highest pairs on the three-octave oblique (which average 0.97). Rivalry incidence drops for the lowest pair of three-octave differences because 4.73 and 0.59 Hz both lie on the low side of the crossover point. Finally, in the two-octave conditions (Figure 3C), the two upper frequency pairs span the 6 Hz crossover point and elicit high rivalry incidence (averaging 0.88) while the two lower frequency pairs do not. In sum, rivalry occurs when the stimuli are sufficiently different to activate separate temporal channels.

Finally, using relative dominance as an index of stimulus strength (Levelt, 1965), the pilot experiment showed that effective stimulus strength tended to increase with temporal frequency (Figure 2). Yet, even when the high-frequency stimulus had maximum contrast, its tendency to predominate was not particularly strong, peaking at about 1.5:1 against the 4.73 Hz stimulus and was not large enough to need correction through contrast adjustment. The reason why the high-frequency modulation predominated more over the 4.73 Hz modulation than the lower rates is not clear. One possibility is that mechanisms signaling static form may also be able to track slow modulations, adding strength to the low temporal channel's response. Overall, however, the lack of strongly skewed predominances confirms that any failures to report rivalry alternations in the 15 s observation period were not due to a strongly dominant pattern assuming dominance for the entire observation period. We therefore presented all stimuli in the following experiments at maximum contrast.

The dynamics of temporal frequency rivalry were not formally measured in this experiment (see Experiment 4 for alternation dynamics), however, observers' subjective experiences were that differences of three or four octaves produced robust rivalry alternations that were typical of those elicited by large (static) orientation differences, with perceptual alternations occurring crisply every one to two seconds. Two octave differences rivaled well if the frequencies were both high, but if both were low rivalry was slow in the manner of rivalry between low contrast stimuli. Frequency differences of one octave seldom produced perceptual alternations, and did not exceed the level of alternations produced by the static control conditions. The control condition, however, probably overestimated the contribution of phase-related rivalry because the phase differences were presented for the entire 15 s observation period, whereas in the temporal frequency rivalry conditions the phase relationship was cyclic, moving in and out of phase periodically.

In sum, temporal frequency rivalry does occur when frequencies differ by two octaves or more, and the control data show that this cannot be attributed solely to periodic phase differences.

## Experiment 2

Experiment 1 established that interocular differences in temporal frequency do elicit reliable rivalry alternations, provided the frequencies differ by at least two octaves. Experiment 2A will measure increment thresholds for the temporal frequencies used in Experiment 1 to verify that perceptual alternations between temporal frequencies with a one-octave difference would have been perceptible. Experiment 2B is a temporal frequency matching experiment that quantifies what frequency is perceived when the temporal frequency difference is too small to produce rivalry. One possibility is that the two frequencies merge into an average and are perceived as an intermediate frequency. One possibility is that a difference frequency or “beat” will be perceived. O'shea and Blake (1986) reported that interocular differences in full-field flicker rates produced a phenomenon similar to a temporal beat pattern at the difference frequency. Carlson and He (2000) also reported a temporal beat of about 2 Hz when LEDs modulating at 28 and 30 Hz were dichoptically presented.

### Methods: Experiment 2A

**Figure 4:**
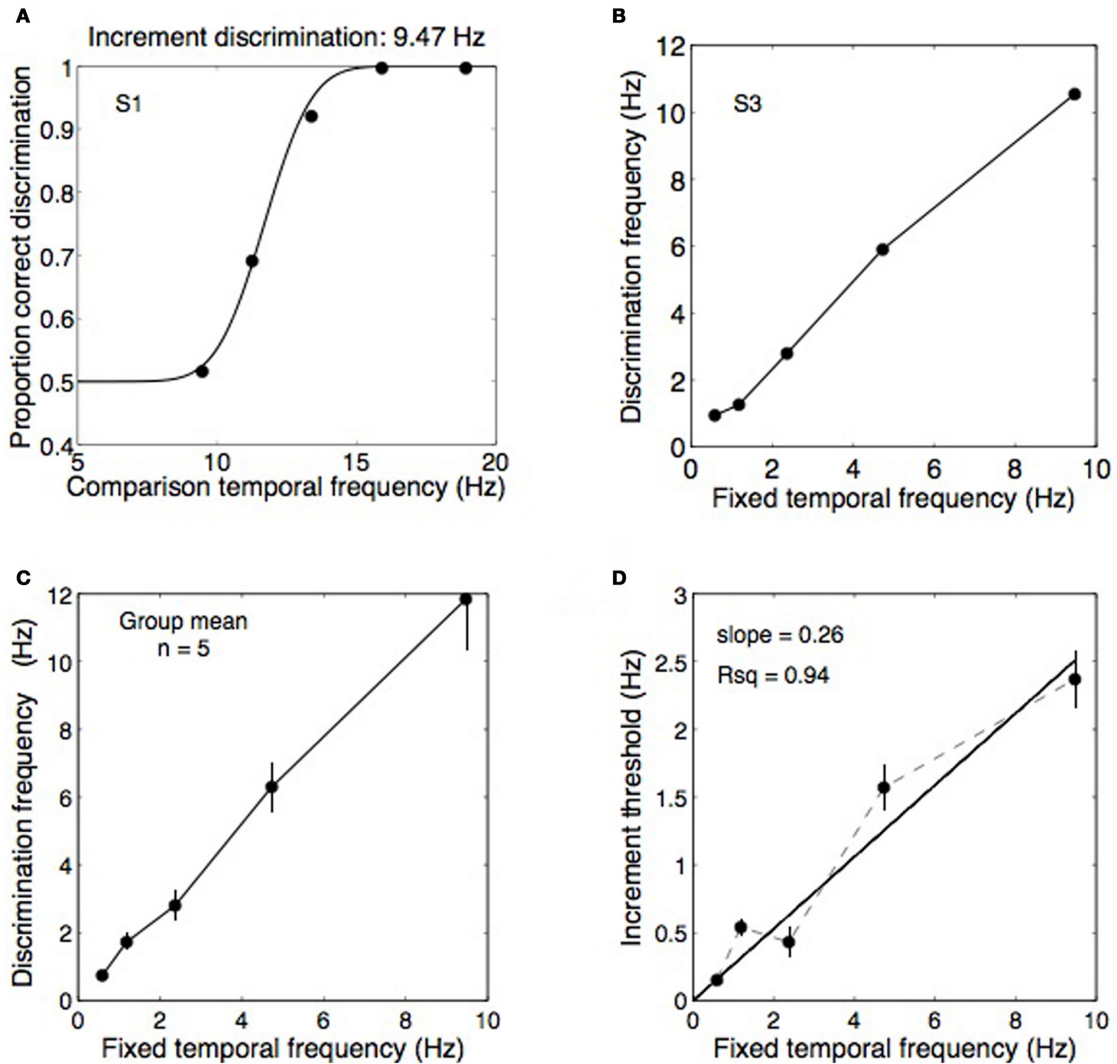
**Data from Experiment 2A showing temporal frequency discrimination performance for a range of base frequencies. (A)** An example psychometric function from one observer discriminating temporal frequency increments on a 9.47 Hz standard. In this example, threshold performance was obtained at a frequency of 11.7 Hz. **(B,C)** Thresholds obtained as in panel **(A)** were measured for five standard frequencies: 0.59, 1.18, 2.37, 4.73, and 9.47 Hz and the data from five observers was pooled into a group mean. **(D)** From the group mean data, the discrimination frequencies were converted to Weber fractions (i.e., Δ*f*/*f*) were computed by plotting the difference between the fixed frequencies and the increment threshold frequencies against the fixed frequencies. The slope of the best-fitting straight line (constrained to pass through the origin) provides an estimate of the Weber fraction, in this case relatively high at 0.26. Error bars show ±1 standard error of the mean.

Spatially, the stimuli were as described in the “General Methods” but the temporal filtering was more narrowly spaced to produce enough resolution for a psychometric function of temporal frequency increment perception. Increment thresholds were measured for all but the highest frequency used in Experiment 1 (0.59, 1.18, 2.37, 4.73, and 9.47 Hz) with the stimuli binocularly presented through a mirror stereoscope. Five observers participated in a two-interval forced-choice temporal frequency discrimination task. Each interval lasted for 2 s separated by a 0.8 s break. In a completely randomized order, each standard frequency was paired with all of its comparison frequencies (see Table 2) a total of 20 times, with the interval order also randomized. Observers indicated which interval appeared to modulate at a higher rate. Psychometric functions were fitted to the data and the frequency increment producing 75% correct performance was taken as the increment threshold (see Figure 4A).

**Table 2:** **A summary of the temporal frequencies of the rivalry stimuli used in Experiment 2B (left-hand side: all are one-octave pairs) and the corresponding nine comparison stimuli for each rivalry pair**.

**Rivalry frequencies (Hz)**	**Comparison frequencies (Hz)**								
0.59 vs. 1.18	0.42	**0.59**	0.84	**1.18**	1.67	2.37	3.35	4.73	6.69
1.18 vs. 2.37	0.42	0.59	0.84	**1.18**	1.67	**2.37**	3.35	4.73	6.69
2.37 vs. 4.73	1.67	1.99	**2.37**	2.81	3.35	3.98	**4.73**	5.63	6.69
4.73 vs. 9.47	3.35	3.98	**4.73**	5.63	6.69	7.96	**9.47**	11.26	13.39
9.47 vs. 18.93	6.69	7.96	**9.47**	11.26	13.39	15.92	**18.93**	22.51	26.77

The rival frequencies within the comparison series are shown in bold.

### Methods: Experiment 2B

Experiment 2B is a temporal frequency matching experiment. The one-octave rivalry stimuli used in Experiment 1 (Table 1, dark oblique) were presented for a random period of between 4 and 8 s followed by an array of nine comparison stimuli modulating a various frequencies. Observers made an unspeeded selection of the comparison closest to the perceived modulation rate when the rivalry period terminated. Table 2 shows the temporal frequencies of each pair of rival stimuli, together with their nine comparison frequencies spaced in quarter-octave steps (half-octave steps for the two slowest modulation pairs). Five observers made 50 matches each to the rivalry pairs shown in Table 2 to produce distributions of frequency matching responses for each pair of one-octave frequency differences.

### Results and discussion: Experiment 2A

Figure 4A shows data from one observer discriminating temporal frequency increments against a 9.47 Hz standard, producing an increment threshold of 11.7 Hz in this case. Thresholds were obtained for five fixed frequencies (Figure 4B) from five observers and were combined into a group mean (Figure 4C). Weber fractions were computed by calculating the differences between the fixed frequencies and the increment threshold frequencies and plotting these differences against the fixed frequencies (Figure 4D). The Weber fractions were well fit by a straight line passing through the origin with a slope of 0.26, confirming that Weber's law holds for temporal frequency discrimination. A Weber fraction of 26% is relatively high relative to other perceptual dimensions. The Weber fraction for spatial frequency discrimination is between 0.08 and 0.13 for a frequency of 1 cpd (Hirsch and Hylton, 1982; Regan et al., 1982), similar to the mean frequency of 1.1 cpd used here, and is 0.15 for speed (Mandriota et al., 1962). Discrimination thresholds for orientation are also very fine, ~1° (Bradley and Skottun, 1984; Bowne, 1990). The magnitude of the Weber fraction may reflect the resolution of the underlying channels. Temporal frequency channels are fewer and broader than orientation and spatial frequency channels (Graham, 1972; Stromeyer and Julesz, 1972; Braddick et al., 1978; Graham, 1989). In any case, a Weber fraction of 26% for discriminating temporal frequencies means that rivalry between two temporal frequencies one octave apart (i.e., 100% as a proportionate difference) should have produced easily discriminable alternations in Experiment 1 if they did elicit perceptual alternations. The fact that alternations were rarely reported for a one-octave frequency difference (Figure 3B) confirms the difference was too small to elicit binocular rivalry.

### Results and discussion: Experiment 2B

**Figure 5:**
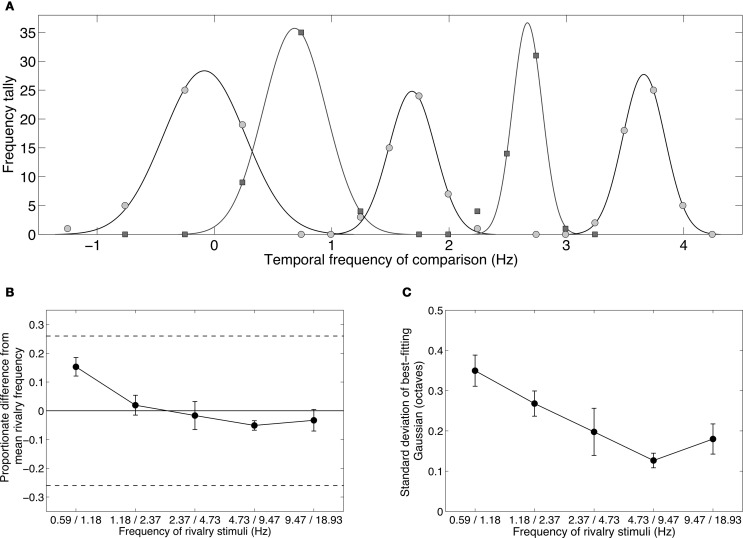
**Results from Experiment 2B showing the temporal frequency perceived during binocular rivalry between stimuli differing by one octave.** Observers chose their matches from a range of nine temporally modulating stimuli (see Table 2). **(A)** Data from one observer showing distributions of temporal frequency matches for each of the one-octave pairs shown in Table 1. The data were well described by Gaussian distributions. Note that the frequencies were converted to a log_2_ scale (i.e., an octave scale) before fitting the Gaussian. **(B)** Group mean data plotting the means of the Gaussian distributions with respect to the geometric means of the rivalry stimuli. A value of zero would indicate a Gaussian distribution centered exactly on the geometric average of the rivalry stimuli. All distributions are very close to this zero point, and all lie within the dashed lines indicating the just-noticeable difference for temporal frequency discrimination. **(C)** Group mean data plotting the standard deviations of the Gaussian distributions. All standard deviations are well less than 0.5, which would correspond to a distribution with a one-octave full-width at half height. All error bars show ±1 standard error of the mean.

Figure 5A shows distributions of temporal frequency matches for the five one-octave rivalry conditions. In each case the distributions are unimodal, as expected if the two eyes were not engaging in binocular rivalry. (Rivalry would produce a bimodal distribution with peaks at the rival frequencies.) The five distributions are separated by about one octave, agreeing with the spacing of the five conditions. Notably, each peak sits approximately halfway between the frequencies presented to each eye, consistent with fusion rather than rivalry. Figure 5B compares the geometric mean (or logarithmic midpoint) of the rivalry frequencies with the distribution peak and shows very little discrepancy: none of the differences exceed the ±0.26 Weber fraction or “just-noticeable difference” (dashed lines). Distribution peaks at the mean of the rival stimuli is consistent with both frequencies activating the same temporal channel, producing an average frequency percept. There was a tendency for the low frequency pair to be perceived slightly higher than their mean, and for higher frequency pairs to be perceived slightly below their mean. This may be due to temporal frequency adaptation, as Johnston et al. (2006) have shown that adaptation to high temporal frequencies lowers perceived frequency, and adaptation to low frequencies raises perceived frequency.

The fact that the distributions of temporal frequency matches were centered tightly around the mean of the dichoptic frequencies shows that observers did not perceive temporal beats, even though the frequencies were too close to elicit rivalry. If temporal beating had occurred, it would have been at the difference frequency. In all five conditions, the modulation rate in one eye was simply twice the rate in the other so the difference would always be equal to the lower of the two frequencies. In none of the conditions were the distributions centered on the lower frequency. Instead, the data point to a perceptual fusion produced by two slightly different frequencies activating the same temporal channel.

Figure 5C plots the standard deviations of the Gaussian distributions. These were all narrow, fullwidths less than one octave, and therefore contained within the one-octave interval between the rivalry stimuli. Lower frequencies produced broader distributions, which might reflect the shape of the low frequency channel, which is broad and low-pass.

## Experiment 3

Experiment 3 uses a temporal frequency matching approach to reveal what frequencies observers perceive when presented with dichoptic frequencies differing by three octaves (1.18 vs. 9.47 Hz), a difference which produced robust perceptual alternations in Experiment 1. The present experiment will confirm which frequencies are perceived and whether there is any bias, perhaps to the higher frequency (O'shea and Blake, 1986). Unlike Experiment 2, frequency matching distributions in Experiment 3 should be bimodal with peaks corresponding to the frequencies of the rival stimuli.

### Methods

Four observers participated in a frequency matching experiment similar to Experiment 2B. The dichoptic temporal frequencies were 1.18 vs. 9.47 Hz and produced strong rivalry. Because of the three octave frequency range, we provided 13 comparison stimuli spaced in half-octave intervals: 0.42, 0.59, 0.84, 1.18, 1.67, 2.37, 3.35, 4.73, 6.69, 9.47, 13.39, 18.93, and 26.78 Hz. Subjects viewed the rival stimuli for brief period (random within 4–8 s) and then chose the comparison frequency most closely matching their percept when the rivalry period ended. Each subject did 75 trials.

### Results and discussion

**Figure 6:**
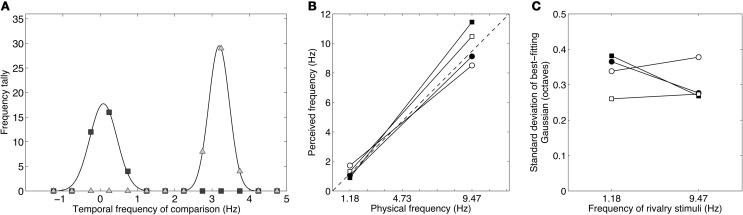
**Results from Experiment 3 showing the distributions of perceived temporal frequency during binocular rivalry between stimuli differing by three octaves (1.18 and 9.47 Hz). (A)** Data from one observer showing a bimodal distribution of temporal frequency matches. The peaks of the best-fitting Gaussians lie very close to the true values of the rivalry stimuli and the data are clearly dichotomous, with no intermediate or “average” frequencies reported. **(B)** Taking the peaks of the Gaussian distributions as estimates of perceived frequency, it is clear that the perceived frequencies correlate very well with the actual frequencies. The group mean peak frequencies were 1.25 and 9.89 Hz. **(C)** The standard deviations of the Gaussian distributions were similar across the group. All standard deviations were well less than 0.5 (i.e., well less than one-octave full-width at half height), indicating that subjects experienced clear rivalry alternations between the competing temporal frequencies and did not experience intermediate frequencies.

Figure 6A shows raw data for one observer. The data are very clearly bimodal, forming two clear distributions with no overlap between them. This confirms the subjective impression when viewing these stimuli that they produced vigorous binocular rivalry with clearly defined alternations between the high- and low-frequency patterns. The two peaks align very closely with the true modulation rates of 1.18 and 9.47 Hz. This was consistent across the group, as shown in Figure 6B which plots perceived frequency (the mean of the Gaussian distribution) against true frequency. The dashed line at 45° is the identity line and the four observers' data clusters closely around it. The peak frequencies across the group averaged 1.25 and 9.89 Hz, very close to the true frequencies of 1.18 and 9.47 Hz, which conforms that subjects did experience rivalry alternations between the competing stimuli. Notably, unlike in Experiment 2B, no matches were made to intermediate frequencies or to the average frequency and the distributions were narrowly distributed around the true peaks. This is shown by the standard deviations in Figure 6C which are much less than 0.5 (corresponding to a one octave fullwidth) and are similar to those in Figure 5C.

Given that rivalry dominance periods are stochastic in terms of duration, (Fox and Herrmann, 1967; Levelt, 1967; Hupe and Rubin, 2003; Brascamp et al., 2005), rivalry between two temporal modulations of equal stimulus strength would mean the final percept in each 4–8 s trial would be unpredictable. This would result in roughly equal numbers of matches to each stimulus, yet the numbers of observations in each distribution were not equal, indicating a bias for one stimulus to predominate more than the other. The low-frequency distribution, when summed across observers, totalled 136 observations, whereas the the high-frequency distribution totalled 164 observations. The biased 40–60% split between low and high frequencies points to slightly greater stimulus strength for the high frequency modulation. This confirms an earlier report of a high-frequency bias with orthogonal counterphasing gratings (O'shea and Blake, 1986), although our bias is somewhat weaker than this earlier report. The high frequency bias is consistent with the recent finding that low temporal frequencies are attenuated by the presence of high frequencies (Cass and Alais, 2006; Cass et al., 2009a) and with the high frequency bias seen in the pilot experiment.

## Experiment 4

The results of Experiments 2B and 3 demonstrate that interocular differences in temporal frequency between spatially matched patterns do elicit rivalry alternations. Temporal frequency rivalry should therefore exhibit the well-known signature of binocular rivalry dynamics with distributions of dominance durations showing a positive skew such as a Gamma distribution (Fox and Herrmann, 1967; Levelt, 1967) or the log-normal distribution (Murata et al., 2003; Brascamp et al., 2005). Also, autocorrelations of rivalry time series should reveal little or no correlation between the durations of successive rivalry periods (Fox and Herrmann, 1967; Levelt, 1967). Experiment 4 aims to verify these two features for temporal frequency rivalry.

### Methods

Five subjects monitored their rivalry alternations while viewing a four–octave temporal frequency difference (1.18 vs. 18.93 Hz) in 10 one-minute trials. Each observer's data were binned into 150 ms epochs and the frequency tallies were normalized to the maximum tally and fitted with a log normal distribution. The resulting frequency histogram was fitted with a log-normal distribution. Autocorrelations were calculated for each observer on the unbinned time-series data.

### Results and discussion

**Figure 7:**
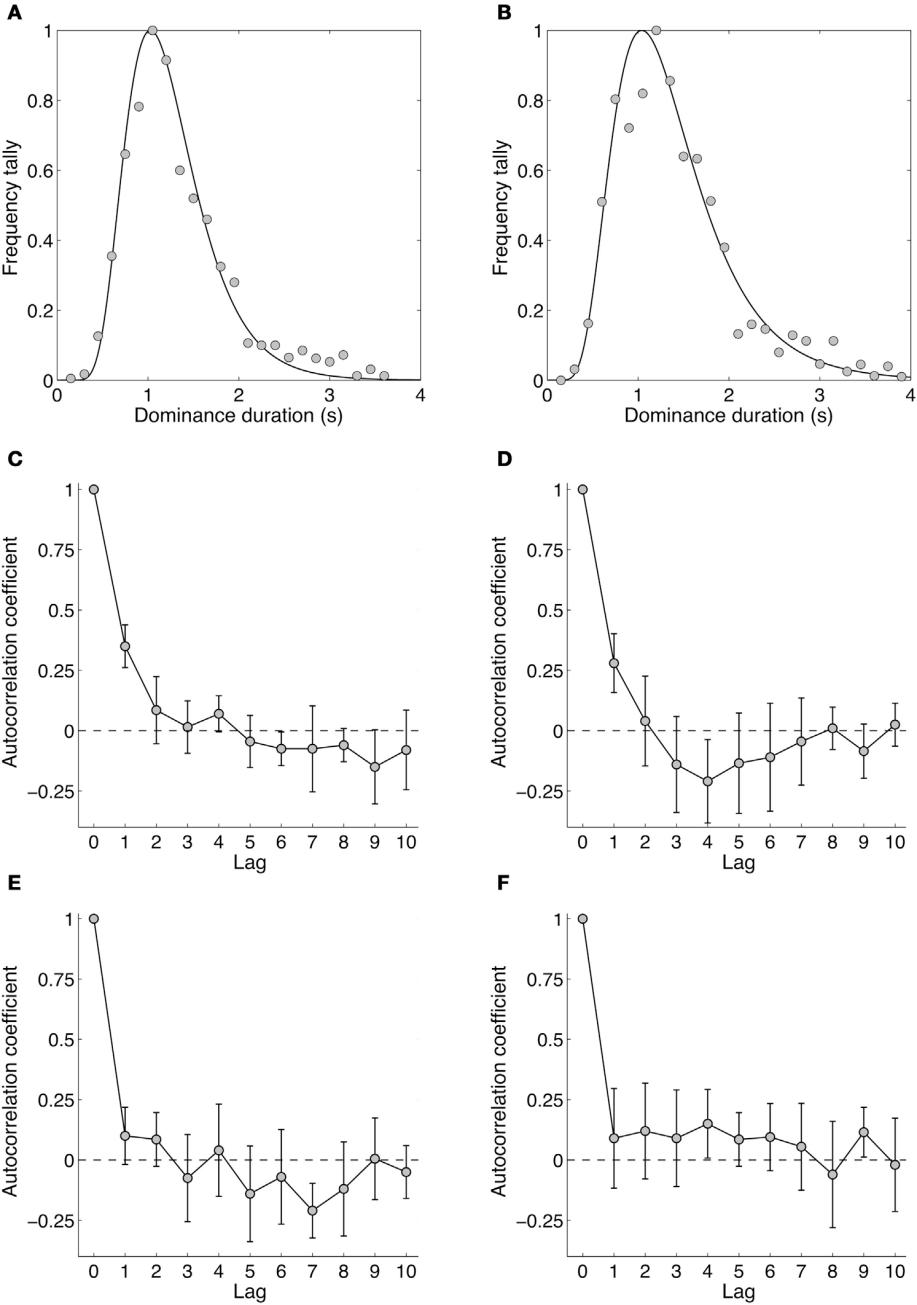
**Data from Experiment 4 showing the dynamics of binocular rivalry between stimuli differing by four octaves (1.18 and 18.93 Hz). (A,B)** Data from two individual observers (authors David Alais and Amanda Parker) showing distributions of dominance times obtained from 10 one-minute rivalry trials. Data were binned into 150 ms epochs and the frequencies normalized to the maximum. The curve shows the best fitting log-normal distribution. **(C–F)** Autocorrelation functions for four observers. Autocorrelations were computed for each of the 10 rivalry periods and averaged. The data in panels **(C–F)** are plotted with 95% confidence intervals, meaning that there are significant lag-one correlations in panels (**C,D)**.

Figures 7A and B show dominance duration distributions from two experienced observers. Gamma distributions traditionally have been fitted to dominance distributions for binocular rivalry (Fox and Herrmann, 1967; Levelt, 1967) and other bistable stimuli (Borsellino et al., 1972; Hupe and Rubin, 2003; Long and Toppino, 2004; Zhou et al., 2004; van Ee, 2005), although the log-normal provides a slightly better description of the distribution (Hupe and Rubin, 2003; Brascamp et al., 2005). Apart from this, the log normal's parameters are more intuitive as they correspond to the distribution's peak dominance duration and its width (i.e., standard deviation) rather than the shape and scale parameter of the Gamma distribution (Brascamp et al., 2005). Overall, the dominance durations from all observers were similar, with a group mean peak duration of 1.29 s and standard deviation of 0.40 s. Overall, the distribution data for temporal frequency rivalry resemble very closely those for spatial rivalry.

Figures 7C–F shows the autocorrelation coefficients for each observer and shows whether the duration of a given dominance period is correlated with subsequent periods. Such analyses often show non-significant correlations for all non-zero lags (Fox and Herrmann, 1967; Levelt, 1967), meaning the durations of dominance percepts are sequentially independent. This is often considered one of the hallmarks of binocular rivalry and these data show that it holds for temporal frequency rivalry as it does for spatially induced rivalry. One notable point is that two of four observers showed significant correlations at lag one, meaning the duration of a given rivalry period was related to the previous one, and other reports too have noted significant lag one correlations (Lehky, 1988; van Ee, 2009). This could arise from neural adaptation operating within a mutual inhibition model of rivalry (Sugie, 1982; Lehky, 1988; Klink et al., 2008; Alais et al., 2010) simply because a long dominance period of one stimulus would lead to more adaptation than would a short period, with a consequently longer recovery period during which the other stimulus would be stronger. This could lead to significant correlations at lag one, as is sometimes observed. Other possible contributions to significant correlations at lag one have been suggested, including attention, eye movements and blinks (van Ee, 2009).

## General discussion

The present study found that interocular temporal frequency differences do produce binocular rivalry, provided the frequency difference is two or more octaves, and that temporal frequency rivalry dynamics show the same characteristics as rivalry induced by spatial differences. Because binocular rivalry is the default outcome when binocular matching fails (Blake and Boothroyd, 1985), these results indicate that temporal frequency is one of the stimulus attributes the visual system uses to decide whether images from corresponding retinal locations are from the same object or not.

**Figure 8:**
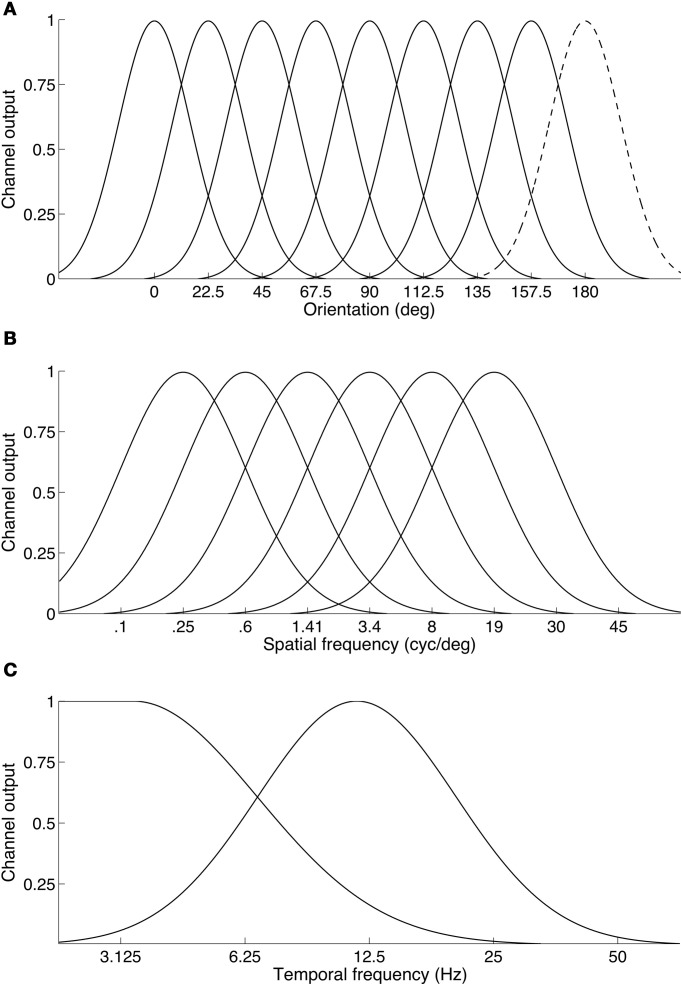
**Illustration of the differing organization of channels across basic visual feature dimensions. (A)** Orientation channels are thought to be narrow and finely sample the orientation dimension. Here eight channels are shown. Because a full cycle of orientation is 180°, the dashed curve centered at 180° is simply a duplicate of the channel located at 0°. **(B)** Spatial frequency channels: six channels are sufficient to span the spatial frequency dimension. The channels shown here have a standard deviation of 1.25 octaves. On a log frequency axis, the channels are modeled as Gaussian normal curves. **(C)** Temporal frequency channels: consistent with many studies, two channels are shown on a log temporal frequency axis, a broad low-pass channel and a high bandpass channel. Some studies suggest a third, very high bandpass channel may also exist.

The results also indicate that binocular rivalry takes place between temporal channels rather than within them. The observation that a two-octave frequency difference (i.e., a four-fold difference, such as 3 Hz vs. 12 Hz) is required to produce robust rivalry supports this because temporal channels are much more broadly tuned and fewer in number than spatial frequency or orientation channels (see Figure 8). Psychophysical studies show the entire temporal dimension is encoded by just two (Anderson and Burr, 1985; Hess and Snowden, 1992; Snowden et al., 1995; Cass and Alais, 2006), or perhaps three temporal frequency channels (Mandler and Makous, 1984; Hess and Snowden, 1992; Johnston and Clifford, 1995). If rivalry is indeed a between channels process it would require rather large temporal frequency differences so that each eye's signal could drive separate channels. Otherwise, frequencies close enough to activate the same channel would merge into an average and binocular fusion would result. This can be seen in Figure 5A where a one-octave temporal frequency difference produced a unimodal distribution of perceived frequencies centered on the average frequency, whereas as larger frequency differences produced bimodal distributions (Figure 6A).

The principle of “rivalry between channels” appears to operate in other stimulus dimensions, even though they are encoded by a finer array of channels. About six narrowly tuned band-pass filters with a full bandwidth of about 1.25 octaves account for our fine spatial frequency sensitivity (Graham, 1972; Stromeyer and Julesz, 1972). About eight tightly tuned band-pass filters with a full bandwidth of 15° underlie orientation perception (Movshon and Blakemore, 1973; Phillips and Wilson, 1984). Because of this finer grain, binocular rivalry can be produced with small spatial differences. For example, compared to the two-octave difference required to trigger temporal frequency rivalry, a one-octave spatial frequency difference will trigger rivalry between two vertical gratings (Blakemore, 1970; Yang et al., 1992). A spatial channel-based approach was also used successfully by Mayhew and Frisby (1976) to account for their study of rivalry and stereopsis. Regarding orientation, orthogonally oriented gratings are a standard rival stimulus yet rivalry can be evoked by orientation differences down to ±15° or less (Braddick et al., 1978). Thus, the interocular differences required to trigger rivalry varies consistently with the grain of the underlying sensory channels.

Another observation indicating that rivalry occurs between channels is that the temporal, spatial and orientation dimensions produce fused percepts when the interocular difference is less than a channel width. Near-vertical lines differing in orientation by a small amount do not rival and instead fuse into a single vertical grating perceived to slant in depth around the horizontal axis, as originally observed by Wheatstone (1838). Similarly, vertical gratings differing slightly in spatial frequency do not rival but fuse into a single grating tilted in depth around the vertical axis (Blakemore, 1970). These percepts are thought to occur when the two stimuli activate a single spatial or orientation channel (Blakemore, 1970; Schor, 1977; Yang et al., 1992) and the perceived depth is an ecologically valid resolution of the small interocular differences. Thus, a consistent principle holds: rivalry occurs when dichoptic signals drive different channels, and fusion occurs when they drive the same channel.

The data in Table 1 reveal a close correspondence between rivalry incidence at various frequencies and the shape of temporal channels. The data on the two-octave diagonal show that it is not the magnitude of the frequency difference *per se* that produces rivalry but where on the temporal frequency dimension the stimuli are located. The two lowest pairs (0.59 vs. 2.37 Hz; and 1.18 vs. 4.73 Hz) produce modest levels of rivalry, whereas the two highest pairs (2.37 vs. 9.47 Hz; and 4.73 vs. 18.93 Hz) produce robust rivalry. This fits with “rivalry between channels” because the cross-over point between the broad low-pass temporal channel and the high bandpass channel is about 6–8 Hz (Anderson and Burr, 1985; Hammett and Smith, 1992; Hess and Snowden, 1992; Snowden et al., 1995; Cass and Alais, 2006). Therefore, the two highest pairs had one frequency on each side of the cross-over, activating separate temporal channels and rivaling strongly. The two lowest pairs had a lower incidence of rivalry because both frequencies strongly activated the low-pass channel, with a modest response from the overlapping portion of the high frequency channel (Figure 8). The absence of rivalry when both stimuli drive the same channel also explains Carlson and He's (2000) report that dichoptic flicker at 28 and 30 Hz produces a temporal beat at 2 Hz, rather than rivalry alternations. Another study examining small dichoptic temporal frequency differences (Baitch and Levi, 1989) compared several frequency pairs (12 and 14 Hz; 18 and 20 Hz; 30 and 32 Hz) and also found reliable 2 Hz beat patterns.

One study arguing against “rivalry between channels” examined dichoptically overlaid translating motion patterns (random pixel arrays) that moved either orthogonally or in opposite directions at various speeds (van de Grind et al., 2001). When slow moving patterns (0, 1.05 and 4.2 deg/s) were paired with patterns moving at speeds of up to 12 deg/s, binocular rivalry was frequently reported, but pairing a slow moving pattern with a very fast moving pattern produced motion transparency. The authors' interpretation was that two speed channels exist—one slow and one fast—and that rivalry occurs when both stimuli activate the same channel (otherwise, transparency results). Two factors might explain this discrepancy. First, they manipulated speed and did not consider the temporal and spatial frequency components of speed (speed = TF/SF) (Smith, 1987; Smith and Edgar, 1991; Alais et al., 2005). Understanding how their stimulus would activate spatial and temporal channels leads to a critical point: their random-pixel stimulus was spatially very broadband, meaning it had a correspondingly large range of temporal frequencies (TF = speed × SF). Consequently, with so much interocular conflict across all spatial and temporal channels, their data could also be interpreted as rivalry between spatial and temporal channels.

There has been a broader debate about whether motion rivalry *per se* exists. Some have argued that rivalry is fundamentally a spatial process resulting from pattern conflict and must therefore occur within the parvo (or form) stream (Ramachandran, 1991; Carlson and He, 2004; He et al., 2005). One study suggesting rivalry does not occur between motion (Ramachandran, 1991) adapted different motion aftereffect directions in each eye but did not observe rivalry between the aftereffects on a static test pattern. Instead they fused into a single direction (Riggs and Day, 1980; Alais et al., 1994). Subsequently, Blake et al. (1998) repeated the experiment and found that conflicting motion aftereffects do produce rivalry alternations, provided a dynamic test stimulus is used. A static pattern, unlike a dynamic pattern, would not effectively tap the adapted state of the MT neurons thought to underlie the MAE (Huk et al., 2001) as these motion-specialized neurons have no sustained response to static patterns.

Another argument against motion rivalry is that motion stimuli invariably contain form and the form conflict triggers rivalry (He et al., 2005). Motion stimuli usually do contain form, whether complex objects or simple gratings, but even stimuli with no coherent form such as translating dots, if moving fast, can leave a pattern of elongated motion streaks due to temporal integration in neurons (Geisler, 1999; Burr and Ross, 2002). Although motion streaks are not usually perceived, they do activate orientation-tuned neurons to induce tilt illusions and aftereffects (Apthorp and Alais, 2009; Apthorp et al., 2010). In a binocular rivalry study, it was shown that “streaks” from fast moving dot patterns produce orientation-tuned rivalry suppression (Apthorp et al., 2009; Stuit et al., 2009), even though no orientation is present in the static stimulus. In some cases, then, apparent examples of motion rivalry may indeed be cases of spatially-triggered rivalry.

It is worth considering whether motion streaks are present in the stimuli we have used here, potentially triggering rivalry from spatial conflict. Geisler (1999) established that dots begin to leave motion streaks once they translate further than their spatial period in a time period of 100 ms (that is, 10 periods per second). Could our temporally filtered stimuli leave motion streaks, creating a source of spatial conflict? Although our stimuli are not translating smoothly in a fixed direction, they do contain specific spatial and temporal frequencies and so speeds can be calculated from the ratio of temporal to spatial frequency: [ 0.59, 1.18, 2.37, 4.73, 9.47, and 18.93] Hz/1.13 cyc/deg = [ 0.5, 1.0, 2.1, 4.2, 8.4, and 16.8 ] deg/s. Given that our stimuli have a spatial period of 0.89°, and the streak threshold is 10 periods per second, the highest temporal frequency used here (18.9 Hz) clearly contains a speed above the threshold to produce motion streaks. This analysis indicates that motion streaks are not likely to have played a role in most conditions in this study, although they may have contributed a spatial component in conditions involving the highest temporal frequency.

Taken together, our results demonstrate that rivalry can occur between temporal frequencies, despite carefully controlled spatial parameters. As temporal frequency channels encode dynamic stimuli, they are part of the magno pathway and our findings show that rivalry is not limited to processes encoding static form. Indeed, our rival stimuli contained very little that would drive cells in the parvo stream because they were unoriented and filtered into a low spatial pass-band (0.8–1.6 cpd). In addition, most temporal modulations in this study were well above the temporal preference of the parvo stream. Our stimuli therefore would strongly activate the magno stream (Lennie, 1980; Gegenfurtner and Hawken, 1996) and yet still elicited robust binocular rivalry, suggesting it is not limited to form conflict.

A related recent paper by Denison and Silver (2012) used flicker-and-swap rivalry (Logothetis et al., 1996) to study magno and parvo processing in binocular rivalry. Flicker-and-swap rivalry can produce slow, irregular alternations (interocularly grouped percepts) and percepts of fast orientation alternations (eye-based percepts). Conditions favoring the magno processing (fast flicker, low spatial frequency) produced more percepts of fast orientation alternation than conditions favoring the parvo processing (slow flicker, high spatial frequency, isoluminance). This implies the motion and form pathways can each engage in rivalry, and each uses a different kind of rivalry process to resolve ambiguous inputs. Carney et al. (1987) also examined form and motion in rivalry using counterphasing gratings with a 90° interocular phase lag. Interocularly grouping these gratings produces smooth motion, whereas a single eye sees ambiguous motion. To induce rivalry, one grating was red/green, the other was black/yellow. They observed color rivalry with unimpaired translational motion, demonstrating color/motion independence in rivalry: suppression of one eye's color does not entail suppression of its motion signal.

## Conclusion

Overall, these experiments demonstrate that interocular temporal frequency differences do produce rivalry in spatially matched patterns. The data can be explained in terms of “rivalry between channels,” with interocularly conflicting inputs to different temporal channels triggering rivalry in the same way that rivalry between orientations and spatial frequencies can be explained. The temporal frequency differences required to trigger rivalry are rather large (about 2 octaves), but are entirely consistent with the broader width of temporal channels relative to the width of orientation and spatial frequency channels. Once triggered, temporal frequency rivalry exhibits the same pattern of temporal dynamics as spatially triggered rivalry. Our results, like those of Blake et al. (1998), provide no support for the claim that binocular rivalry is exclusively a parvo-pathway function, and are consistent with earlier work showing that motion and form rivalry are independent (Andrews and Blakemore, 1999; Alais and Parker, 2006).

### Conflict of interest statement

The authors declare that the research was conducted in the absence of any commercial or financial relationships that could be construed as a potential conflict of interest.
